# Supplementary material for: Venomix: a simple bioinformatic pipeline for identifying and characterizing toxin gene candidates from transcriptomic data
Source: PeerJ. 2018 Jul 31;6:e5361. doi: 10.7717/peerj.5361 (PMC6074769; doi:10.7717/peerj.5361)
Supplement: Supplemental Information 6 [file peerj-06-5361-s006.gz › FinalOutput_1E-6/Alpha-latroinsectotoxin-Lh1a_1/finaltree.pdf]

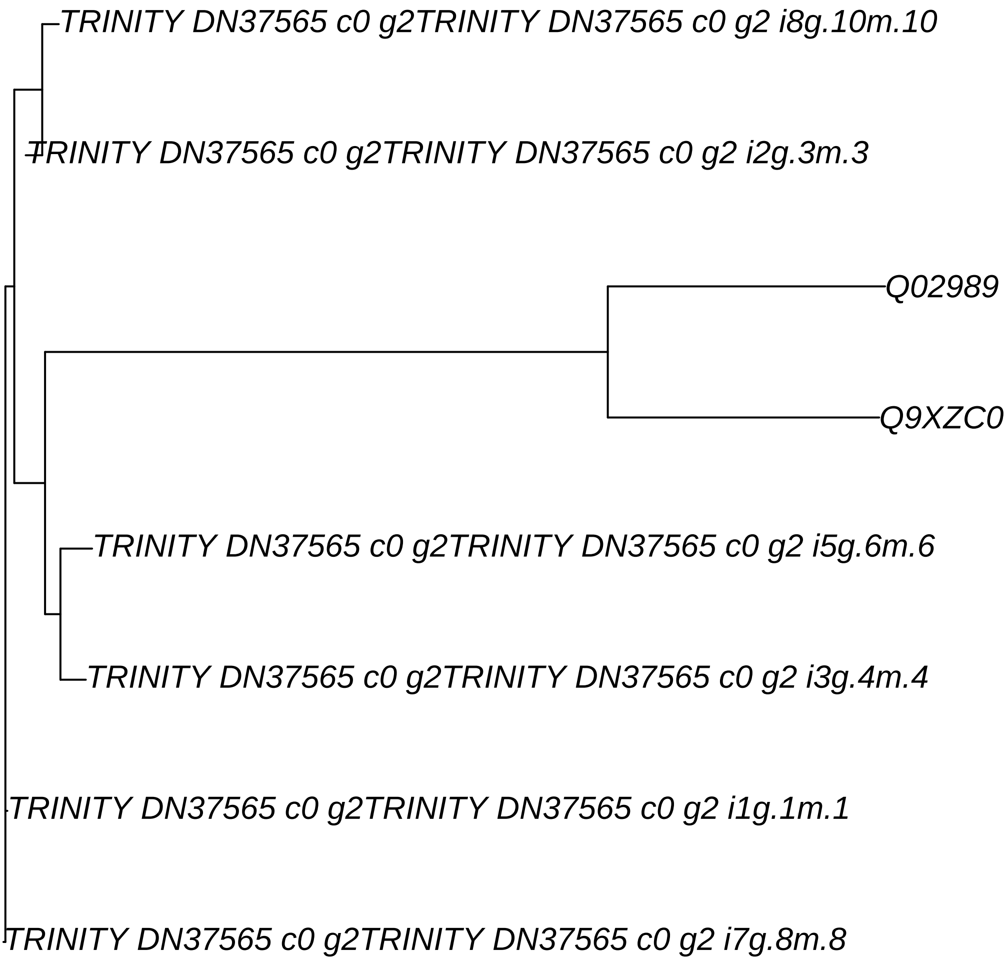

TRINITY DN37565 c0 g2TRINITY DN37565 c0 g2 i8g.10m.10
TRINITY DN37565 c0 g2TRINITY DN37565 c0 g2 i2g.3m.3
Q02989
Q9XZC0
TRINITY DN37565 c0 g2TRINITY DN37565 c0 g2 i5g.6m.6
TRINITY DN37565 c0 g2TRINITY DN37565 c0 g2 i3g.4m.4
TRINITY DN37565 c0 g2TRINITY DN37565 c0 g2 i1g.1m.1
TRINITY DN37565 c0 g2TRINITY DN37565 c0 g2 i7g.8m.8
